# Extracts

**Published:** 1884-01

**Authors:** 


ATLANTA

### Medical Register.

Vol. III.] JANUARY, 1884. [No. 4

### Extracts.

### TREATMENT OF PUERPERAL SEPTICÆMIA.

Let us now suppose that, in spite of every precaution, the specific poison has gained entrance at one of the numerous door-ways left open in the genital tract between the vulva and the fundus uteri; what are the most reliable means now known to us for checking the advance of the septic disorders which are set up in consequence?

But let me stop here, before answering the question just asked, and explain what I mean by the use of the term specific poison. I do not believe that there is, necessarily, any specific disease germ which gives rise to *puerperal* septicæmia! It is probably the same germ as that which is the source of septicæmia, phlebitis and lymphangitis in the stump after an amputation, in the wound created by a compound fracture, or in the lacerated tract produced by a gun-shot. But the pathological condition excited appears to me to be entirely different from that putrid absorption which results from the decomposition of a retained placenta, or a putrid mass of blood. Such decomposition produces a toxæmia, violent and dangerous it is true, but which disappears as soon as the offending mass is removed. That of the true puerperal disease at once, or almost at once, diseases the lymphatics and sets up an action which often proves uncontrollable. If the mere presence of decaying animal material in a uterus would produce puerperal septicæmia without the agency of a specific disease germ, we should surely have that affection developed in healthy country localities where the women are attended by ignorant midwives, but where, nevertheless, it is almost an unknown disorder.

"I," says Hervieus, "who write these lines, declare that in my own country I have, within the space of three years, attended one thousand cases of labor, and out of that number have lost only one patient!"

And now, in summing up what I esteem the most certain and the most rational treatment of the disease styled puerperal fever, I will be as concise as possible.

As soon as the patient is stricken by the poison certain very marked phenomena usually develop themselves with great promptness. After a chill or a slight horripilation she is affected by a high temperature, pelvic pain, considerable mental purturbation, headache, pain in the back, and sometimes, though not commonly, by nausea and vomiting. We will assume, first, that the attack is a severe one in its inception; and second, that the patient is in such a position in life that we are not in any way hampered in our efforts to save her by considerations of economy. Having considered treatment from these standpoints, it will, of course, be easy to modify the plan so as to meet the requirements of a mild attack or of a scanty purse.

As the practitioner sits by the bedside of his patient at the commencement of her attack, he is aware that there are points connected with its true pathology which he cannot yet determine. For example, he cannot say whether the case is going to assume the form of septicæmia lymphatica or septicæmia venosa; whether of perimetritis or parametritis; or whether thrombosis of some of the large pelvic or utero ovarian vessels, or a true parenchymatous metritis is to play the most active part in the siege which has begun. If he fritter away the golden moments in vague speculation; if he soothe his fears by hoping that the attack is due to malaria or milk fever; or, if he cast aside the rational doctrines of to-day in favor of the idea of a general infectious and particular form of disease called by the forefathers of the French school "*la fievre des femmes en couche*," time will be lost which can never be regained. If, on the other hand, he is encouraged by his clinical observation to stand with many of the best pathologists and practitioners of our time in the position assumed by Hervieux—"I believe in the multiplicity of the affections classed under the head of puerperal fever; I believe in puerperal poisoning as the source of them;—he will act at once and strike at the poison before it has fairly gained a foothold. In other words, if the physician could see into the future and learn with certainty that peritonitis, cellulitis, thrombosis, lymphangitis, or true phlebitis is to be the final disorder, he should, if he reaches the case at the inception of the attack, follow, in my opinion, the course here formulated:

1. As soon as a diagnosis of septicæmia is determined upon, all pain, nervous perturbation, shock and mental anxiety should be quieted by the hypodermic administration of ten minims of Magendie's solution of morphine, unless some special and very decided idiosyncrasy with reference to opium be ascertained to exist; and throughout the severity of the attack, whenever suffering of mind or body occurs (perhaps it will be about once in every six or eight hours), this should be repeated. In my experience no other method of administering morphine in these particular cases compares with this, and, as it is not to be continued long, there is no fear of causing the patient to become addicted to the drug as a vice. If a small, sharp and *new* needle be used, if it be thoroughly cleansed with soap and water before each time of using, and be dipped in a solution of bichloride, 1 to 1,000 of water, just before each insertion, no abscesses will occur. It is the large, rusty, unwashed and unpurified needle, which the doctor's economy makes last him for many months, which so commonly results in them.

2. The physician must now decide whether, in his opinion, the septic disease which is developing has originated in the wounds situated between the os internum uteri and the vulva, or in the endometrium, above the former point. If he decide in favor of the former view, he should persist, for a time longer, in the more thorough use of vaginal injections; if of the latter, intra-uterine injections should be at once resorted to. Usually the question has to be settled by the efficacy or inefficacy of frequent germicide vaginal injections in bringing down the temperature and controlling other grave symptoms. Should a failure of these seem to prove that the origin of the disease is higher up the general tract, more decided and radical measures must be taken.

The patient having been entirely relieved of pain and thoroughly quieted, the first injection should be practiced in this way: An india-rubber cloth should quietly, without, hurry, noise or disturbance on the part of the nurse, be spread over the edge of the bed on which she lies, and made to fall into a tub of warm water rendered antiseptic by the addition of 2 or 2½ per cent. of carbolic acid, or of the bichloride of mercury, 1 to 2,000, or of some other reliable germicide. Then Chamberlain's glass uterine tube, which I here show, or the very excellent and ingenious tube invented by Dr. George H. Lyman, which is here seen, thoroughly fitted to a Davidson's or Higginson's syringe, should be immersed in the tub. The nurse now aiding the patient by the shoulders, and the doctor by the hips, she should be gently laid across the bed and be made comfortable with a pillow under her head. Each foot should rest upon a chair placed at either side of the tub, and she should be entirely covered over with a couple of blankets. The doctor, now placing himself between the knees of the patient, should take the tube in his right hand, while a stream of water is made to flow through it by the nurse, who squeezes the syringe bulb; and he should pass it gently up to the fundus of the uterus. The stream of water, which has been steadily flowing, is now projected with gentle force against the walls of the uterus, washing away adherent blood-clots, detaching portions of hanging membrane, and everywhere neutralizing the influence of the poison which has excited the disorder.

After the first injection, the position of the patient need not be disturbed, but the injection may be given as she lies upon a bed-pan.

In some cases, in which I have had reason to suspect that portions of the placenta or membranes have been retained, I have chloroformed the patient, passed the hand, rendered thoroughly aseptic, within the cavity, and very gently scraped off adherent masses from the uterine walls, using the nails as a curette, as Wilson, of Baltimore, advised. In some other cases I have rubbed the whole endometrium with an asceptic sponge, held in a long sponge-holder, or employed the largest of my curettes, to remove clots and adherent secundines, with great apparent advantage.

That the use of antiseptic uterine injections after parturition is attended by danger, is beyond question. The greatest hazard attending this plan is the entrance of air into the uterine cavity; the next, the production of hemorrhage by detaching some of the thrombi which fill the mouths of the uterine sinuses; the third, the danger of forcing the fluid used as an antiseptic directly into the general circulation, through the introduction of the tube into the mouth of a sinus; the fourth, the creation of convulsions, violent pain, or nervous prostration, by a sudden and baneful influence upon the nervous system; and the fifth, the passage of the tube into a Fallopian canal, and the injection of fluid directly into the peritoneal cavity, as in a case reported by Dr. W. Gill Wylie in an interesting paper in the *New York Medical Journal* for June 23, 1883, p. 679.

All these dangers may be, to a great extent, avoided by care as to details: by using a large injecting tube which can not enter an open-mouthed sinus; by using water warmed to 105°; by injecting the fluid through the tube so as to exclude air before passing this up to the os uteri; by using only a moderate degree of force in throwing the jet against the uterine walls; and by proceeding with the whole affair gently; cautiously, slowly and intelligently.

The tube should never be allowed to fill the os uteri completely, so as to prevent the escape of the injected fluid. Should the cervical canal be so narrow as to hug the tube closely, it should be dilated by dilators of hard rubber, by the fingers, or by Barnes' bags, before the injection is practiced.

A solution of the persulphate of iron should always be at hand in case of sudden hemorrhage from displacement of a thrombus. Should this accident occur, ergot should be immediately given hypodermically, the iron solution be at once added to the antiseptic solution and allowed to pass into the uterus, and pressure be made upon the fundus so as to stimulate the contraction of uterine fibre to accomplish closure of the open sinuses in that way.

Quite a number of cases of death from this plan of treatment are on record. In a very large experience with it I have met with but one. The whole number on record would, however, fall, I think, into insignificance if weighed in the balance against the many deaths which have been due to a neglect of the means, or against those lives which have been saved by it.

Aiter all, the question as to the dangers attending a plan of treatment are not to be settled upon mere abstract reasoning. The evil which it is known to do must be weighed in one scale, and the good which it affects in another; and careful consideration must decide whether we are justified in accepting the former for the sake of the latter. Judged in this manner, I feel very sure that intra-uterine injections for puerperal septicæmia deserve a place among the most valuable resources for the saving of life for which we are indebted to modern pathology.

The frequency of these intra-uterine injections should vary greatly with individual cases. In mild cases of septicæmia, where the temperture comes readily down after the uterus has been washed out, and rises very slowly, they need only be used once in every five hours; in other cases they become necessary once in every three hours; and in bad cases they are required once every hour. These injections should always be administered by a physician, should always be carried fully up to the fundus uteri, and should always be used with every regard to caution as to detail which has been already mentioned.

Many prefer the use of those syringes which allow of a steady flow of a stream of water, propelled by gravitation, as is the case with the so-called fountain syringe, which is so popular among us. This is partly because greater safty is supposed to attach to these. and partly from a theory that danger attends the propulsion of a stream by intermittent jet against the uterine walls. For a number of years I shared this belief, but experience has taught me that a gentle projection of the fluid is an advantage, that by this means a more thorough cleansing is accomplished, and that with due caution no more danger attends the plan than that by the steady flow

Some have adopted continuos irrigation of the uterine cavity, but this is, I feel perfectly certain, a delusion and a snare. It gives the appearance of great thoroughness, which it does not possess, for the reason that by this plan it is very difficult to bring the germicide fluid into full contact with the entire endometrium. For vaginal irigation it is an excellent method, but I have seen it allow the temperature to remain high when applied to the uterine cavity, and have replaced it by the intermittent douche used only as often as every three hours, with striking results. Nevertheless, in very severe cases I prefer to employ continuos irrigation, replacing its use every third hour by that of the intermittent current; rather than exhaust my patient by half-hour disturbances and injections, as has been by some advised.

After all that has been said on this subject, the essential fact is this: that plan is best which accomplishes most perfectly the cleansing of the parturient canal. With ordinary precautions, danger need not necessarily attach to any method.

3. The uterus having now been thorougnly cleansed, and the patient entirely quieted, attention should be turned to controlling the temperature, which, in septicæmia of puerperal character, runs so high and maintains itself at so exalted a range as to constitute one of the immediate factors of a fatal issue. Even if this were not the case, the patient's strength is so much exhausted by prolonged high temperature, her nerve power so much depreciated, her blood-state so rapidly injured, and her comfort so decidedly interfered with, that these considerations alone would point to the propriety of combating hyperpyrexia. For this purpose I formerly relied upon the affusion of cold, or tepid water, the patient lying upon Kibbee's cot; at present I accomplish the same result more easily and more pleasantly for the patient by the use of Chamberlain's rubber-tube coil, which I here show. A mat, composed of a rubber tube rolled upon itself in a circle, covers the whole abdomen from the ensiform cartilage to the symphysis pubis; the upper end of the tube which makes this mat is anchored by a weight in a tub of ice-water, placed about three feet above the level of the patient, and the lower end falls into a tub upon the floor. By siphon action the water of the elevated tub runs through the tube which constitutes the mat, aud collects in the receptacle on the floor. By this means a temperature of 104° can very readily, as a rule—for there are exceptions to the rule—be kept at 100° for weeks together.

Unfortunately, there are almost insurmountable difficulties connected with the use of this invaluable method in the minds of the patient's friends, the patient herself, the nurse, and alas, too often, the attending physician. You are told that the patient becomes chilled, that the coil prevents her resting, that the temperature absolutely goes up under its use, and descends whenever it is left off; and by the doctor you are apt to be informed that his fear of resulting pleurisy, bronchitis, or pneumonia is very great.

I will merely say, in refutation of these charges, that in my service in the Woman's Hospital, where convalescents from laparotomy are constantly under treatment in large numbers, this means of controlling temperature is as commonly and as freely in use as poultices are in general hospitals, or gargles in dispensaries for diseases of the throat. We never meet with any of these difficulties, and very rarely with failures as to the desired result, and I believe that I am correct in saying that successive house-staffs, whose duty it has been to carry out the plan, have thus far had, to a man, the most implicit faith in its beneficial agency.

There are some peculiarities about it, however, which I must mention. Very often the coil will not succeed in controlling the temperature for twenty-four hours; its prolonged use alone develops and illustrates its great benefits; and removing it from the body for an hour at a time damages its influence very much. I have never seen evil result from the chilliness which it excites, if hot bottles be kept at the soles of the feet, and in not one instance, out of hundreds of cases, have I seen pneumonia or pleurisy excited by it.

4. The nervous system should be so kept under the influence of febrifuge medicines as to keep under control the tendency to chill the pyrexia. For this purpose, fifteen grains of the sulphate of quinine should be given in capsule or by suppository night and morning, or, in place of this, two capsules may be given night and morning, of Warburg's tincture, in the form of solid extract, as advised by Dr. J. T. Metcalfe. Lastly, to the same end the salicylate of sodium may be employed.

5. The patient's diet should consist entirely of fluid food, given often and in small amounts. The staple article should be milk, but animal broths and gruels may be alternated with it with advantage.

6. At the very commencement of such a case the attending physician should, in the patient's interest, surround himself with efficient and abundant assistance. If he undertake to wash out the uterus every four or five hours without other assistance than that of the nurse; and if the patient is to rely for the constant attention and care which she will inevitably require, upon one nurse, it is needless to point out that the duty of both doctor and nurse—vital duties, be it remembered—will be but half performed. And, unfortunately, the penalty will fall upon the patient and her friends.

For a case of puerperal septicæmia to be properly treated in private practice by the plan here advocated, it is necessary for the attending physician to associate with himself some young practitioner, who has time to devote to the case, and the intelligence to use the uterine douche safely and efficiently. Furthermore, two nurses are necessary; one to be in charge for twelve hours, and the other then to relieve and replace her for an equal time. Without the rest and sleep thus afforded, no nurse taking charge of such exacting cases as these can possibly do her duty in such a way as to subserve the patient's interest. As a rule, the nurse will begin with the declaration that she is competent and willing to take entire charge; that she is one of those people who can do without sleep, etc.; and her heroic offers of self sacrifice are hailed with enthusiasm by the terrified family. To believe in, and to act upon, this would be very foolish and very dangerous. After three nights of watching, this same nurse would snore through the hours in which her patient needed her, give the wrong medicines, mislead the doctor, allow the bladder to become distended with urine, and fail in every requirement of the occasion.

If the patient cannot bear the great expense attendant upon the extra service which I have mentioned, it is her misfortune; and for such as herself the doors of our hospitals are open. There is no case of ovariotomy in the Woman's Hospital, however poor the patient be, upon which, thanks to the generous arrangements of its managers, just such attendance as I have mentioned is not lavished.

The antiseptics which have heretofore been tried under these circumstances are thymol, boric acid, salicylic acid, carbolic acid, and mercuric bichloride. Of these, all have disappeared before the superior merits of the last two; and carbolic acid, which for so long a time has been almost supreme, appears about to be abolished in favor of the bichloride of mercury, 1 part to 1,000 or 2,000 of water. For all antiseptic purposes outside of the uterus, the bichloride is now, owing to the carefully made and important investigations of Koch, very generally employed in the strength of 1 to 1,000, and the uterus has now been washed out with this excellent germicide, 1 to 2,000, often enough to make us regard its use as an intra-uterine injection as entirely warrantable. If carbolic acid be used in that way, it will not be safe to carry its strength beyond a two, or, at most, a two-and-a-half-per cent.

---

Danger of Poulticing Eyes.—*C. M. Hobby, M.D, in Iowa State Medical Reporter.*—It is a melancholy fact that more eyes are destroyed by poultices in this State than by all forms of traumatism. The common resort of the household practitioner, too often prescribed by those who have had opportunities to know better, the poultice continues to add a not inconsiderable proportion to the annual increase of blindness.

The efficiency of warmth and moisture in relieving the pain of local inflammation is not doubted; that it promotes the softening of abscesses, and that it favors the migration of leucocytes are well know, but the very reasons for its efficiency in general surgery are contra indications for its employment in diseases of the eye, and forbid its use in certain forms of disease of the ear.

The application of moist heat in certain corneal troubles, in cyclitis, and even the continuous use of warmth and moisture when an eye has been destroyed, and orbital cellulitis exists, are allowable, but unless the eye has lost its function irreparably, hot fomentations should be employed only under the watchful care of the surgeon, and then only for an hour or so at a time.

In the light of recent experience I am inclined to believe that it is better to do without them under all circumstances, than to risk ihe possible mischief that may result, from the patient's recommending their use to others.

I make no distinction between poultices, from the decomposing bread and milk, the dirty-looking flaxseed, the potato, alum, curd, rotten apples, or even cow-dung poultices, to the more elegant warm water, muslin, and oil silk, all by furnishing warmth and moisture favor the migration of leucocytes into the bloodless tissue of the cornea, the production of corneal abscess, and the train of mischief following from corneal suppuration, and terminating in iritic adhesions or pan-opthalmitis.

The principal danger in purulent conjunctivitis, aside from the fact that nature in the swollen and oedematous lids, in the increased temperature, and the discharge from the conjunctional surface, furnishes the best kind of a poultice, which rapidly causes corneal infiltration. Again, under the influence of warmth and moisture the vascularity of the conjunctiva is increased, its papillae enlarged, cell proliferation favored, and a simple, acute conjunctivitis becomes rapidly converted into a chronic catarrhal, more intractable than a trachoma, and indeed, seldom recovering until true granulations are formed. During the last summer I have seen three cases of slight traumatism of the cornea terminate in sloughing of the entire anterior layers of the cornea, two of them in pan-opthalmitis and one in orbital cellulitis, under the influence of vigorous poulticing, and probably not less than twenty cases of corneal abscess induced by poulticing in conjunctival inflammation, and to these I now add a case, in which corneal abscess entirely destroyed the cornea, which, prior to the poulticing, was sound, as was also the conjunctiva. The case was one of traumatism, involving the sclerotic one-third of an inch back of the cornea, caused by a puncture from an awl, which passed through the sclerotic, invading the vitreous, and probably wounding the lens. The lad was free from pain for about a week, and had little if any loss of vision, when he was attacked with pain along the supra orbital and nasal nerve, probably from iritis, accompanied by redness of the eye. A poultice was applied with the effect of relieving the pain, but when first seen by me his cornea was sloughing through its entire extent, the anterior chamber filled with pus, and the lens opaque, with sympathetic irritation of the other eye. While the eye might have been lost if never poulticed, yet as the ciliary region of the globe was uninjured, there existed a reasonable chance of preserving some useful vision without danger to its felly; this chance was lost by the use of poultices. But the eye will not have been sacrificed in vain if the profession will appreciate the fact that a healthy cornea may become invaded with leucocytes under the favoring influence of warmth and moisture.

---

RATTLE-SNAKE POISONING TREATED BY POTASSIUM PERMANGANATE.—Emil Broies, a student of medicine in Bellevue, reports the following in the *Polyclinic:* In the early part of May, 1883, the weather being very warm, a man, aged about 35, was bitten in the calf of the right leg by a rattle-snake. He was stepping close to some loose rocks, and heard the peculiar warning rattle of the snake, but before he could recover himself, was struck. He was taken to his hut by his companion, who first killed the reptile and secured its rattles, which numbered seven, with the button. By the time I reached the patient, four hours had elapsed. I found him very pale, cold sweat covering his face and extremities, pulse slow and irregular. He complained that his sight was failing, and was in great pain. He had vomited several times. I examined the wound and found the leg badly swollen, presenting a very glossy surface of an erysipelatous nature, quite tender to the touch. The point of entrance of the fang was quite pale, while the surrounding surface was of a light red hue. I asked for stimulants, but none could be had. I had read that potassium permanganate had been used with happy results in cobra poisoning; I thought it would be well to try it here. I immediately injected about one fourth of a grain into the wound and the same quantity into the arm, for fear that the solution injected into the wound would not be taken up into the circulation. I also gave him two grains by the mouth. Before leaving him, I arranged matters so that he could receive two grains by the mouth and one-fourth of a grain injected every hour, alternately, but I had little hope for success. I was, indeed, much surprised, on visiting him next morning, to find him looking well, his pulse full and regular, his eyesight good, his appetite fair, and the swelling diminished nearly one-half. However, I made a poultice of meal, mixed with a small amount of aqua ammonia, and told him to take half a drachm of aromatic spirit of ammonia every three hours, continuing the use of the potassium permanganate by the mouth, but discontinuing the injection. On the following day he was much better. I then discontinued all but the permanganate, which was to be taken every three hours, keeping the wound moist with about ten per cent. solution of ammonia water. The fourth day he was out and at work of a light nature.

The following is the most rational explanation and treatment of premature baldness which has come under our notice. We take it from the *Boston Med. and Surg. Journal. The Edinburgh Med. Journal* reproduces from the *Berliner klinische Wochenschrift* (No. 16, 1883), the following note: O. Lassar has continued his observations on the nature of premature baldness, and has further convinced himself of the communicability of at least the form associated with dandruff. When the hairs which fall off in such cases are collected, rubbed up with vasaline, and the ointment so made is rubbed among the fur of rabbits or white mice, baldness rapidly makes itself visible on the parts so treated. That this is not due to the vasaline was shown by anointing other animals with the vasaline alone, which produced no effects whatever. He considers that the disease is spread by hairdressers, who employ combs and brushes to their customers, one after another, without any regular cleansing to these articles after each time they are used. During frequent visits to the hairdressers' it can scarcely fail that brushes are used which have been shortly before dressing the hair of one affected with so common a complaint as scaly baldness. Females, he thinks, are less often affected with this form of baldness, because the hairdresser more frequently attends to them at their own homes, and there uses their combs and brushes. In order to prevent, as far as possible, the commencement of alopecia prematura, the hair should be cut and dressed at one's own home, and with one's own implements, and these thoroughly clean. When it has begun, the following mode of treatment is suggested: The scalp is to be daily well soaped with tar or fluid glycerine potash soap, which is to be rubbed in for fifteen minutes firmly. The head is then to be drenched with, first, warm water, and then gradually colder water. A two per cent corrosive sublimate lotion is next to be pretty freely applied. The head is then to be dried, and the roots of the hair are to have a one half per cent. solution of napthol in spirit rubbed into them. Finally, a pomade of one and a half to two per cent. of carbolic or salycilic oil is to be used to the head. This treatment has now in many cases brought the disease not only to a stand, but the hair has been to a considerable extent restored.

---

Useful Notes on Water.—One gallon of distilled water weighs 10 pounds; one gallon of sea water weighs 10.32 pounds; 1.8 cubic foot of water weighs one hundred weight; 36 cubic feet weigh one ton, equal to 224 gallons; one cubic foot contains 6¼ gallons. (The English standard, or Imperial gallon, is here referred to). The average daily consumption of water in towns is from sixteen to twenty gallons per head. In pipes the square of the diameter in inches equals pounds' weight of water per yard. Example: a 3-inch pipe holds nine pounds per yard. One-hundredth inch of rain is about one ton's weight to the acre. A nominal horse-power for a boiler requires one cubic foot of water per hour. Circular apertures are most effective for discharging water, since they have less frictional surface for the same area. The *vena contracta* is the best form of orifice for discharging water. The ordinary speed to run a pump is from eighty to a hundred feet per minute. The pressure in pounds per square inch of a column of water is the height in feet, multiplied by 594, or, for an approximation, one-half pound pressure per square inch for each foot of height. Water in flowing through an aperture has a velocity equal to that acquired by a heavy body falling freely from a height equal to the distance between the centre of the aperture and the surface of the water. Doubling the diameter of an aperture increases the flow fourfold. A man can raise water from a well ten feet deep at the rate of thirty gallons per minute. The approximate time occupied in discharging equal quantities of water, under equal heads, through pipes of equal length, varies from 80 for a straight pipe, 200 for a curve, to 220 for a right angle.

---

The Transmission of Diptheria from Children to Fowls.—*The Medical Press* contains a synopsis of observations made by Dr. L. Roth, of Kissengen, upon a violent outbreak of diptheria among barnyard fowls, which he attributes to infection from children through poison from these being mixed with the sweepings of the room and thrown into the yard. These observations would tend to establish a similarity between the pip, as occurring in poultry, and diptheria in the human being. This similarity has long been suspected, although as far as our knowledge goes no definite observations have been made looking to a tracing of the similarity in pathological conditions. The deduction, however, from Roth's observations would seem to emphasize the necessity of great care for children against infection during the prevalence of pip in poultry. There may be more in the suggestion than has even been suspected, and these observations may possibly tend to stimulate further research in this direction.

---

Dr. Bartholow's antiseptic treatment of typhoid fever is as follows: Purgative doses of calomel during the first week. This is to regulate heat production, and to destroy typhoid germs in the intestine. One to three drops of a mixture of one part of strong carbolic acid and two parts of tincture of iodine is given every three hours, day and night, throughout the disease.

---

The venerable Dr. J. L. Atlee, the celebrated American ovariotomist, recently mistook an enormously enlarged right lobe of liver for an ovarian tumor. The wound was immediately closed and the patient put to bed. The very best diagnosticians may err occasionally.

---

Professor Erb holds that syphilis is one of the most important, if not the most important cause of the occurrence of tabes. That tabes is a specific disease, a late manifestation of syphilis, he does not consider to be proved though he thinks it extremely probable.

---

Dr. Truzzi vaccinated a number of pregnant women during the last three months of pregnancy to determine if the child would thus be protected. The results were negative, as the children were all successfully vaccinated a few days after birth.

---

A Doctor's Diploma in Court.—The following, from the *Presbyterian Observer*, is said to be a report of an address to the jury of a farmer whom a doctor had sued for services. The late Alexander H. Stephens was attorney for the defense and Robert Toombs for the plaintiff, it appeared so plain that Mr. Stephens advised his client to pay up, and refused to address the jury. The defendant, however, declined to follow this advice, and requesting the privilege of himself addressing the jury, was allowed to do so, and this is the way he did it:

"Gentlemen of the jury, you and I is plain farmers, and if we don't stick together these ere lawyers and doctors will take advantage of us. I ain't no lawyer or doctor and I ain't no objections to them in their proper place, but they ain't farmers, gentlemen of the jury. Now this man, Royston, was no doctor, and I went for him to come and doctor my wife's sore leg, and he came and put some salve truck on to it, and some rags, but never done it a bit of good. Gentlemen of the jury, I don't believe he is a doctor any way. There is doctors as is doctors, sure enough, but this man dont earn his money, and if you send for him, as Mrs. Sarah Atkinson did for a negro boy as worth $1,000, he just kills him and wants you to pay it."

"I don't," thundered the doctor.

"Did you cure him?" asked Peter, with the slow accents of a judge with a black cap on. The doctor was silent, and Peter proceeded:

"As I was saying, gentlemen of the jury, we farmers, when we sell our cotton, has just got to give vally for the money we ask, and doctor's ain't none too good to be put to the same rule. And I don't believe this ere Sam Royston is a doctor no how."

"Look at my diploma, if you think I am no doctor."

"His diploma!" exclaimed the orator, with great contempt. "His diploma! Gentlemen, this is a big word for printed sheepskin, and it don't make no doctor of the sheep as first wore it; nor does it of the man as now carries it; a good newspaper has more in it, and I pint out to ye that he ain't no doctor at all." The doctor was now in a fury, and screamed out :

"Ask my patients if I am not a doctor."

"I asked my wife," retorted Peter. "She said she thought he was not."

"Ask my other patients," said the doctor.

This seemed to be the straw that broke the camel's back ; for Peter replied with look and tone of unutterable sadness : "That is hard saying, gentlemen of the jury, and one that requires me to die, or to have powers as I have heard tell ceased to be exercised since the Apostles. Does he expect me to bring the angel Gabriel down to toot his horn before his time, and cry aloud, 'Awake, ye dead, and tell this court and jury your opinion of Sam Royston's practice ?'

Can I to go to the lonely churchyard and rap on the silent tomb, and say to um as is at last at rest from physic and doctor's bills, 'Get up here, you, and state if you died a natural death, or was you hurried up some by doctors.' He says ask his patients, and gentlemen of the jury, *they are all dead!* Where is Mrs. Beasley's man, Sam ? Go ask the worms in the graveyard, where he lies. Mr. Peak's woman, Sarah, was attended by him, and the funeral was appointed, and he, the doctor, had the corpse ready. Where is the likely Bill, as he belonged to Mr. Mitchell ? Now in glory, expressing his opinion of Royston's doctoring. Where is that baby gal of Harry Stephens ? She is where doctors cease to trouble and the infants are at rest. Gentlemen, he has eaten chicken enough at my house to pay for his salve. I found the rags, and I don't suppose he charges for making her worse, and even he don't pretend to charge for curing her, and I am humbly thankful he never gave her nothing for her inwards, as he did his other patients, for something made them all die mighty sudden."

The applause was great, and it is needless to say the doctor lost and Peter won.

---

Poisonous Ice Cream.—Truly there's a poison-drop in man's purest cup. Death lurks in nooks we know not of, and anon in its rapacity claims its victims. The latest ambuscade which has been revealed is the innocent looking and seductive ice cream, that prominent feature of social gatherings and church festivals, without which these affairs would be stale, flat and unprofitable.

According to *Home Health* about two hundred people who attended a church festival at Joliet, Illinois, were taken after eating ice cream with diarrhœa, vomiting, painful cramps and considerable prostration, although, fortunately, these symptoms were not the forerunners of any serious or fatal consequences. The symptoms were so similar to those caused by tartar emetic, that the local profession gave it as their opinion that this drug had, accidentally or otherwise been added to the cream. A sample of the lot partaken of was taken in charge by the authorities and sent to Dr. Chas. B. Gibson, chemist, to the College of Physicians and Surgeons, of Chicago, for analysis. Dr. Gibson found no antimony, and thus allayed the suspicion of foul play. He, however, detected ptomaines, alkaloids which were formed in the first stages of the decomposition of animal matter, but which disappear or are destroyed by further decomposition.

The process of making the cream in question was favorable to the developments of ptomaines. It appears that after the cream had been collected, it stood three or four hours, when it was subjected to heat, then stood a few hours longer before the freezing. Some of the cream contained the germ which has the power of starting the process of decomposition, and forming the ptomaines. The boiling process put a stop to further decomposition, and thus fixed the poisonous alkaloids, for they could not then be destroyed by further decomposition, and boiling and freezing is not able to destroy them.

Milk, above all other articles of food, is liable to contain impurities from the air. It may contain, and very often does, the spores of every kind of bacterial germ, from the *tubercle bacillus*, the recognized cause of consumption, to the germ of diphtheria and other diseases, Whatever may be floating in the air is very likely to get into milk, and without doubt any sort of germ finds that milk is a very proper foods medium, in which it can live and multiply its race.

The prevention of milk contamination is nearly impossible. The sort of contamination which adulterates milk simply depends upon what may happen to be in the air. The germ which cause the manufacture of ptomaines are not always in the air equally, at all times and places, but when they are, food of any kind, as well as milk, is liable to become contaminated, and then we read the account of poisoning by canned fruit or meat in families, or the poisoning of whole church festivals by ice cream. The different methods of preserving various food in air-tight cans, or by cold or heat, are efficient only because these methods exclude the germs of putrefaction. If fruits or meat are cooked or frozen, the germs are, perhaps, destroyed, and then if the food is inclosed in cans it is more or less protected from germs in the air.

---

ON PERSONAL PRECAUTIONS THAT MAY BE ADOPTED BY MEDICAL MEN WHILST ATTENDING CASES OF INFECTIOUS DISEASE.—Dr. Charles Green makes these suggestions in Lancet:

1. Always have the window open before entering the patient's room or ward.
2. Never stand between the patient and the fire, but always between him and the open window.
3. If possible, change your coat before entering the room.
4. Do not go in for unnecessary auscultation or other physical examination.
5. Stay as short a time as possible in the room.
6. Never, while in the room, swallow any saliva.
7. After leaving the sick-room, wash the hands with water containing an antiseptic.
8. Rinse out the mouth with diluted "toilet Sanitas" or Condy's fluid, also gargle the throat with it, and bathe the eyes, mouth and nostrils.
9. Expectorate and blow the nose immediately on leaving the sick-room.
10. Keep up the general health by good food, exercise and temperance.

11. In additions to the above recommendations, which are all pretty generally known, I would suggest another, which is, in my opinion, the most important of all. This is to filter all the air you breathe while in the sick-room or ward through an antiseptic medium. My method is to use a McKenzie's inhaler over the nose and mouth. I carefully soak the sponge in a strong solution of carbolic acid before entering the sick room. It is so made that all the air breathed must necessarily come through this sponge, and the expired air is emitted by a valve action at another place. I have worn this not only in the Fever Hospital wards, but in many of the typhus dens in this borough. It is to this method that I attribute the fact that although I have attended between 200 and 300 cases of typhus during the last twelve months, and seen many more, I have hitherto escaped infection myself. The only objection (which is not of much importance in a hospital) is the unsightly appearance one has with the inhaler *in situ.* This objection is, however, a very slight one when weighed against the greatly increased safety one not only feels, but, I believe, actually possesses. I am not aware of this method having been mentioned previously; and this fact, and my desire to prevent a repetition of the late disastrous fatalities, must be my apology for bringing it before the profession.

---

A New Treatment of Cancer.—Prof. Nussbaum, whose name is very familiar to the profession in connection with his method of treating ulcers, discusses a new treatment of cancer in the Muenchen *Med. Woch.*, a translation of which appears in the *Physician and Surgeon.* After enumerating the unsuccessful attempts at preventing the development of cancerous tumors by ligating the main artery supplying the part, or by injecting various remedies into the parenchyma of the tumor, or around it, and the frequency of their recurrence after extirpation with the knife, he proposes his new method. He had noticed, in connection with his plan of treating indolent ulcers of the leg, that when an incision was made around them about a finger's width from their margin down to the fascia, the incision being prevented from healing, improvement rapidly set in. He now proposes a treatment essentially similar for cancer. Not long ago he treated a case of cancer of the mamma, with so strong a tendency to hemorrhage that each dressing of the ulcer threatened the woman's life. As the patient was nearly moribund from the frequent loss of blood, he surrounded the tumor with a strong subcutaneous ligature, which he drew together with all his strength and then tied. The result was not only an arrest of the hemorrhage and a speedy rally of the patient, but a constant decrease of the tumor to one-quarter of its size, when the ulcer began to cicatrize. He now proposes to prevent the feeding of these tumors by their peripheral nutrient vessels in the manner indicated. The blood which supplies the tumor through its base, while it does not absolutely starve it, is, however, sufficient to keep it stationary. In order to accomplish the desired interruption of the blood supply from the periphery, he urges the formation of a furrow around the tumor down to the fascia, about one centimetre wide, made by means of a thermo-cautery.

In the case above referred to a tubercle had not been included in the ligature. Nine months after the application of the ligature the tubercle had grown and had thus reached the tumor of the mammæ, which latter had become quite solid. He treated the new growth by surrounding all the isolated tubercles with the deep furrow spoken of, and in six weeks had the satisfaction of seeing all the tubercles shrink in size. Nussbaum thinks the thermo-cautery, or actual cautery, is much more useful than is generally believed. The subject of the condition of the patient, his strength and appetite are much better after an operation with a cautery than with a knife. The wound heals more quickly, except in cases where approximation by sutures can be employed after the operation by the knife. The superior advantages of the cautery are referred to the absence of loss of blood, and to the fact that the nerves and blood vessels, being covered with a cicatrix, are protected from the influence of the air.

---

Preventive Treatment of Mammary Diseases.—Dr. Richard Wood speaks with much confidence in the *Midland Medical Miscellany* of the following plan of treatment by way of preventing the development of mammary diseases:

After a confinement all may go on well for three or four days or more, when the patient may complain of soreness about one of the nipples. Always let us pay immediate attention to her complaint and request; both are warnings to us which must not be disregarded. A glass shield with a tube and an india-rubber nipple for the infant should be at once used. Tannin glycerine applied frequently, and if, notwithstanding these precautions, the mother continues to suffer, the child must be taken from the breast, and probably the pain will subside and all go well, provided there is no tenderness in the substance of the breast, but if tenderness and pain are left in it attended with some appreciable hardness confined to one place, and some redness, our best endeavors must at once be directed to the treatment, and no time must be lost. Should shivering have occurred we may already be too late.

To insure success we should take the case in hand on the same day as that on which the pain and hardness are first noticed.

Upon being called to a case in the early stage, we apply a piece of lint doubled four times, and large enough to cover the neck and tender part of the breast, having previously soaked the lint in one ounce of belladonna liniment, with some gutta percha tissue to cover the whole, and then this is bandaged up carefully with three bandages, each six yards in length. Internally we prescribe gr. x of calomel in a pill and the following mixture:—

R—Tr. belladonnæ f. ʒii, mag. sulph. ℥iss.

Spt. chlorof. ʒii, aq. destill. ad. ℥vj.

Mist. ft. Two tablespoonfuls every three hours.

In the case of very delicate women, we must watch the effect of the medicine and stop it if the bowels act more than four or five times, to be resumed again on the following day; but the belladonna should not be stopped unless the throat, or head, or tongue, or pulse, make it necessary. The next day the dressing should be removed and the lint soaked in more belladonna liniment, and reapplied as before, taking care to support the breast well up by bringing the bandages round the neck and shoulders every time. After about three days the hardness and inflammation should have disappeared, in which case the dressings need only be repeated a few more times every alternate day. All stimulating beverages should be stopped from the first day of treatment, and likewise meat; in fact, for the first day the less she eats the better. At the same time the nurse should have orders not to be afraid to give brandy if there should be signs of fainting in consequence of the violent action of the aperient.

He states it as his firm belief, based on an experience of ten or twelve years, that no breast during lactation would ever suppurate if the above treatment could be adopted early enough. Unfortunately the physician is not called until it is too late—sometimes three days after the hardness and pain were first experienced.

---

A New Tune on an Old String.—The following story is culled from the pages of the California Medical Journal, for October, 1883: "An old toper whose sober moments were harassed by a vixenish wife concluded to shuffle off, and loaded up with laudanum for that purpose. In a short time his wife discovered him in a state of narcotism, and, raising an alarm, sent off every one who came in for a physician. The first one who came was Smith, an old practitioner, who looked him over, pronounced him dead, and went away. Soon after, another old practitioner, Brown, came in, who also gave in the verdict of 'dead,' and departed. Shortly the third one, Jones, a young practitioner, arrived, and, proceeding to a vigorous use of the stomach-pump and forced exercise, finally succeeded in bringing the old gentleman to his senses, and left, feeling that there was but one first-class doctor in that vicinity. In a few days he called around and presented his bill. 'What's this for? inquired the would be suicide. For saving your life the other night,' replied Jones. 'Well, I didn't ask you to. I never employed you, and I'll not pay it. You'd no business coming in here and jamming your old pump down my neck. Brown is my family physician, and I'll not pay any body else,' was answered. Then Jones went away to Brown's office to try to induce the man to pay the bill. 'Jones,' said Brown, looking out over the top of his spectacles, 'I never thought you a bad sort of a fellow, but you've done a very foolish thing, and it serves you right to lose your bill. Didn't I say he was dead? 'Yes,' says Jones. 'Did'nt Smith say he was dead? 'Yes,' says Jones. 'Well, that settled it. The man was dead to all intents and purposes, and you had no right to say that he was not. When two old experienced doctors like Smith and me say a man is dead, it's unprofessional and discourteous for a young man, a beginner in practice, to dispute their word. We'll forgive you this time, because of your youth and inexperience, and will hush the matter up for you; but be very careful in the future and make no more such mistakes.'"

---

A New Remedy for Diphtheria.—It is to Germany that we are indebted for this efficacious and simple treatment, which consists of large doses of oleum terebinthinæ rectificatum. Different writers have highly extolled it and ascribe to it actions which seem almost miraculous.

It would appear that scarcely half an hour after its administration a bright scarlet tint begins to encircle the diphtheritic exudation, gradually increasing in size, at length completely envelope and replaces the false membrane.

Authors, who have been liberal in their praises, affirm that within twenty-four hours after the ingestion of the remedy, the disease has wholly disappeared, leaving hardly a trace.

The treatment, however, appears to be attended with such marvelous success and promptness of effect only when the disease is in the first stages; nevertheless, even after the disease has existed several days, though not as prompt, it exerts a decided curative action and hastens the ultimate recovery. The dose, which is best given immediately after, or in a little warm milk, ranges from a teaspoonful for children to a tablespoonful for adults, morning and evening.—*Journel de Therap.*

A Leech in the Larynx.—It seems remarkable that a leech should establish itself at the base of the epiglottis and be perched upon the arytenoid cartilage without a warning note to arouse the unwilling host. We infer, however, that in the instance recently observed by M. Visusse, such knowledge on the part of the sufferer must have been the case from reading that the laryngoscope was employed to make clear what was the cause of the incessant hæmoptysis, dysphagia and dyspnœa in the case recorded. The mirror revealed the blood-sucker in the situation already indicated. It is satisfactory to know that the patient was relieved of his symptoms and of the leech by means of a pair of Cusco's forceps.—*Lancet.*

---

Treatment of Soft Chancres and of Buboes by Salicylic Acid.—The efficacy of salicylic acid in the treatment of soft chancres and of buboes appears to us to be unquestionable. While not an absolute specific, it is, in our opinion, capable of being most advantageously employed. Odorless, only slightly painful in its application, soluble in alcohol and in glycerine, and leaving no stain on linen, it is preferable, in these important respects, to most other agents employed for the cure of the above named affections, while perhaps inferior in certain other particulars to some among its rivals. It may be resorted to in all cases, and is equally available in private and hospital practice.—*Jour. of Cut. and Ven. Dis.*

---

Cough Mixture.—

Wild cherry bark and senega aa............℥iv.
Ipecacuanha.............................℥ii.
Ex. of conium..........................gr. xv.
Water q. s. ft. (by displacement)......fl. ℥viii.
Then add gin and comp. tr. cardomam aa...℥i.

M. S. Two teaspoonfuls in water, dose to relieve cough.

This originated with Prof. Pancost, and is especially valuable for persons who cannot taste opiates without discomfort.

